# COM-1 Promotes Homologous Recombination during *Caenorhabditis elegans* Meiosis by Antagonizing Ku-Mediated Non-Homologous End Joining

**DOI:** 10.1371/journal.pgen.1003276

**Published:** 2013-02-07

**Authors:** Bennie B. L. G. Lemmens, Nicholas M. Johnson, Marcel Tijsterman

**Affiliations:** Department of Toxicogenetics, Leiden University Medical Center (LUMC), Leiden, The Netherlands; University of California Santa Cruz, United States of America

## Abstract

Successful completion of meiosis requires the induction and faithful repair of DNA double-strand breaks (DSBs). DSBs can be repaired via homologous recombination (HR) or non-homologous end joining (NHEJ), yet only repair via HR can generate the interhomolog crossovers (COs) needed for meiotic chromosome segregation. Here we identify COM-1, the homolog of CtIP/Sae2/Ctp1, as a crucial regulator of DSB repair pathway choice during *Caenorhabditis elegans* gametogenesis. COM-1–deficient germ cells repair meiotic DSBs via the error-prone pathway NHEJ, resulting in a lack of COs, extensive chromosomal aggregation, and near-complete embryonic lethality. In contrast to its yeast counterparts, COM-1 is not required for Spo11 removal and initiation of meiotic DSB repair, but instead promotes meiotic recombination by counteracting the NHEJ complex Ku. In fact, animals defective for both COM-1 and Ku are viable and proficient in CO formation. Further genetic dissection revealed that COM-1 acts parallel to the nuclease EXO-1 to promote interhomolog HR at early pachytene stage of meiotic prophase and thereby safeguards timely CO formation. Both of these nucleases, however, are dispensable for RAD-51 recruitment at late pachytene stage, when homolog-independent repair pathways predominate, suggesting further redundancy and/or temporal regulation of DNA end resection during meiotic prophase. Collectively, our results uncover the potentially lethal properties of NHEJ during meiosis and identify a critical role for COM-1 in NHEJ inhibition and CO assurance in germ cells.

## Author Summary

Sexually reproducing animals create germ cells via meiosis, a cell division program that requires the induction and faithful repair of DNA double-strand breaks (DSBs). Meiotic DSBs are typically repaired via homologous recombination (HR), an error-free repair pathway that generates transient links between homologous chromosomes, named crossovers (COs), which are needed for proper chromosome segregation. To date, it is unclear how germ cells channel these programmed DSBs into HR and not into error-prone DSB repair pathways such as non-homologous end joining (NHEJ). We used the genetically tractable animal model *Caenorhabditis elegans* to study the mechanisms underlying the strong HR bias in germ cells. Here, we identify COM-1, the worm homolog of CtIP, as a crucial regulator of meiotic DSB repair pathway choice: COM-1 effectively blocks the action of the NHEJ complex Ku, thereby assuring correct repair via HR. In addition, we show that unscheduled NHEJ activity during meiosis leads to a lack of COs, extensive chromosomal aggregation, and near-complete embryonic lethality. Further genetic dissection also revealed a redundant and stage-specific role for COM-1 in meiotic HR. Our work thus establishes COM-1/CtIP as a caretaker of germline genome stability and unveils meiotic NHEJ as a potent source of chromosomal aberrations in newborns.

## Introduction

DNA double-strand breaks (DSBs) are toxic DNA lesions that, if not repaired correctly, can cause gross chromosomal alterations. For this reason, DSBs are potent inducers of cell death as well as malignant transformation [1]. Two major DSB repair mechanisms have evolved that are able to repair DSBs: an error-free pathway called homologous recombination (HR) and an efficient but error-prone pathway called non-homologous end joining (NHEJ) [2], [3]. Together NHEJ and HR safeguard genome integrity, however, on a mechanistic level, they are mutually exclusive. NHEJ is based on DNA end protection: the Ku70/Ku80 heterodimer stabilizes the double-strand (ds) DNA ends and prepares the DSB for direct ligation by DNA ligase IV [2]. In contrast, HR is based on DNA end resection: nucleases degrade the dsDNA ends to expose 3′ single strand (ss) DNA tails, which then form a nucleoprotein filament with the recombinase RAD51 that promotes strand invasion and subsequent DNA synthesis reactions [3]. Because of its conservative nature, HR is better suited for maintaining genome stability, but it requires an undamaged DNA template (i.e. the sister chromatid or homologous chromosome), which is not always available. As a result, most human cells (especially non-cycling somatic cells) typically rely on NHEJ for DSB repair [2], [4].

DSB repair fidelity is particularly important in germ cells, as they harbor the genetic material that is passed on to the next generation. Germ cells create haploid gametes via a specialized program of cell division called meiosis, in which a single round of DNA replication is followed by two subsequent rounds of chromosome segregation (named meiosis I and meiosis II). Separation of the parental/homologous chromosomes during meiosis I requires the induction of programmed DSBs [5]. Meiotic DSBs are introduced by SPO11, a highly conserved topoisomerase-like protein that, after cutting, remains covalently bound to the 5′ ends of the DSB. Loss of SPO11 function leads to severe chromosome missegregation and aneuploid gametes in many model systems, highlighting the importance of meiotic DSB formation for successful gametogenesis and species survival [5], [6].

Meiotic DSBs need to be repaired via HR, as only this pathway creates repair products known as crossovers (COs), which are required for the establishment of chiasmata, the transient links between homologous chromosomes. Chiasmata are essential for proper chromosome alignment and segregation during meiosis I [7]. Given that NHEJ competes with HR and does not lead to COs, this activity should be restricted in order to assure chromosome stability during gametogenesis. Previous studies have revealed that *Caenorhabditis elegans* (*C. elegans*) germ cells posses NHEJ activity, yet in wild-type germ cells this error-prone pathway seems to be inhibited very efficiently [8], [9], [10], [11].

Recent insights on mitotic DSB repair have led to the identification of several factors that are able to block NHEJ activity, including the tumor suppressor CtIP [12]. Studies on DSB repair pathway choice in meiotic cells are hampered by the fact that crucial regulators like CtIP are required for mammalian development, which precludes analysis of CtIP-deficient gametes [13]. Here, we exploited the *C. elegans* model system to explore if the CtIP homolog COM-1 is responsible for the robust HR bias in metazoan germ cells; maternal contribution of *com-1* gene products enables *com-1* mutant embryos to develop into adults that produce COM-1-deficient germ cells [14]. COM-1/CtIP is well conserved throughout evolution and homologous counterparts named Sae2 and Ctp1 have also been identified in the unicellular organisms *S. cerevisiae* and *S. pombe*, respectively [14], [15], [16]. In yeast, Sae2/Ctp1 is required for Spo11 removal and therefore is crucial for the initiation of meiotic DSB repair [17], [18].

Here, we show that COM-1 is dispensable for meiotic recombination *per se*, yet it is crucial to complete meiosis: COM-1 is required to block toxic Ku activity at meiotic DSBs and therefore is needed to prevent chromosome aggregation and CO failure. In addition, we reveal a role for COM-1 in interhomolog HR: COM-1 acts parallel to the nuclease EXO-1 to generate RAD-51-coated recombination intermediates at early/mid pachytene stage. We thus identified a dual role for COM-1 during metazoan meiosis: it blocks toxic NHEJ activity and guarantees the timely formation of interhomolog COs.

## Results

### COM-1–deficient germ cells bear chromosomal aggregates and univalents

**Figure 1:**
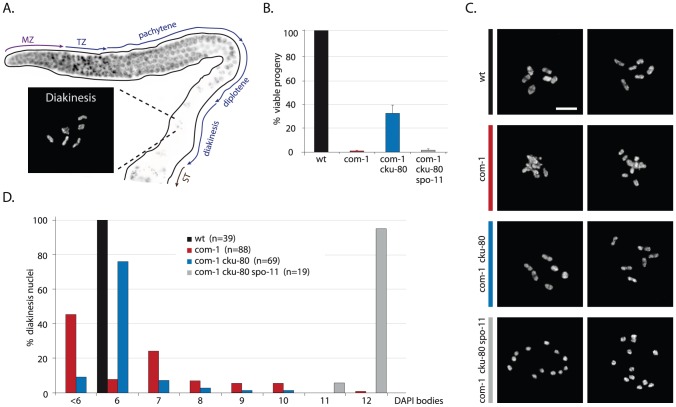
Loss of *cku-80* prevents chromosomal aggregation and restores chiasmata formation and embryonic survival in *com-1(t1626)* mutants. (A) Schematic overview of the *C. elegans* germline, in which different zones correspond to the successive stages of meiotic prophase. MZ: mitotic zone; TZ: transition zone; ST: spermatheca. Blow-up shows a typical wild-type diakinesis nucleus with six bivalents. (B) Percentage progeny survival of animals of the indicated genotype; values are the average of 3 independent experiments, error bars represent S.E.M. (C) Two representative pictures of diakinesis nuclei of animals of the indicated genotype (D) Frequency distribution of DAPI-stained entities at diakinesis. n = number of germlines analyzed. Scale bars, 5 µm.

**Figure 2:**
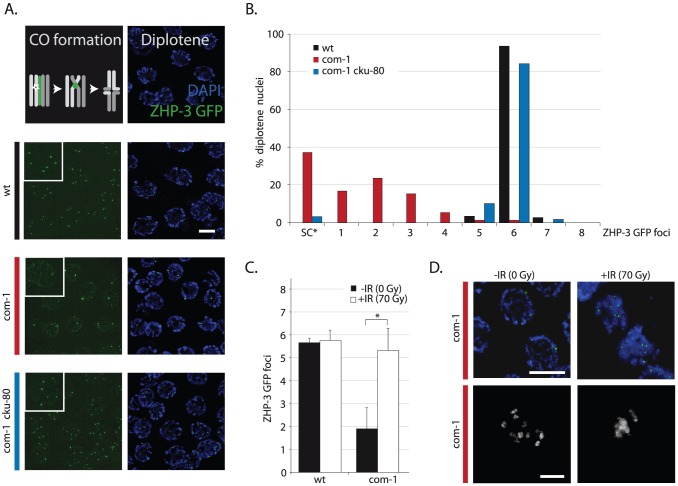
Loss of *cku-80* as well as γ-irradiation rescues the CO defect of *com-1* mutants. (A) Localization pattern of ZHP-3::GFP at diplotene stage. Upper panel shows schematic overview of dynamic ZHP-3 re-localization (green) during CO formation; lower panels show representative pictures of diplotene nuclei of animals of the indicated genotype that express a ZHP-3::GFP transgene (left: GFP signal only, inset = blow-up of single nucleus; right: merge of GFP and DAPI signal). (B) Frequency distribution of ZHP-3::GFP foci in diplotene nuclei of animals of the indicated genotype; SC* = ZHP-3::GFP signal along the synaptonemal complex, no distinct foci (C) Average number of ZHP-3::GFP foci in diplotene nuclei of wild-type or *com-1* mutant animals 24 hours after mock/IR-treatment: 0 Gy (black bars)/70 Gy (white bars); Error bars represent SD, *the difference between mock- and IR-treated *com-1* mutants was highly significant (p<0.001 by Student's t-test, two tailed) (D) Upper panel: representative pictures of diplotene nuclei of mock/IR-treated *com-1* mutants that express a ZHP-3::GFP transgene (merge of GFP and DAPI signal); Lower panel: representative pictures of diakinesis nuclei of mock/IR-treated *com-1* mutants that express a ZHP-3::GFP transgene (DAPI signal only). Scale bars, 5 µm.

**Figure 3:**
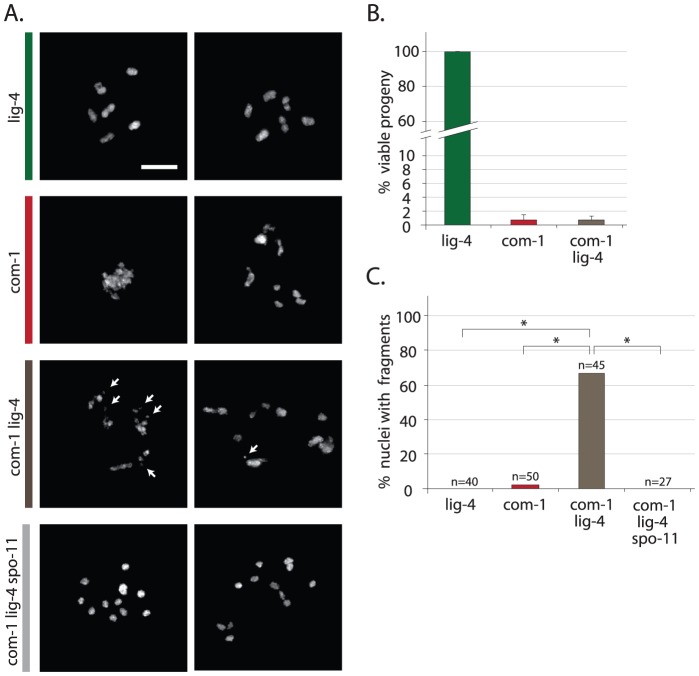
Loss of *lig-4* prevents chromosomal fusion in *com-1* mutants, but does not restore viability. (A) Two representative pictures of diakinesis nuclei of animals of the indicated genotype. White arrows point out chromosomal fragments (B) Percentage progeny survival; values are the average of 3 independent experiments, error bars represent S.E.M. (C) Percentage of diakinesis nuclei that show chromosomal fragments; n = number of germlines analyzed. Scale bars, 5 µm. *The difference between these genotypes was highly significant (p<0.001 by Fisher's exact test, two tailed).

In order to study the meiotic functions of COM-1 we obtained two different *com-1* mutant alleles previously identified by Penkner and colleagues (Figure S1) and [14]. In *C. elegans*, defects in repair of meiotic DSBs can be detected relatively easily, as these often manifest as chromosomal abnormalities in diakinesis nuclei of maturing oocytes (Figure 1A). Wild-type diakinesis nuclei typically have six rod-shaped DAPI-stained bodies named bivalents, which represent the six pairs of homologous chromosomes, each held together by chiasmata (Figure 1A and Figure 2A). In the absence of meiotic DSBs (e.g. in *spo-11* mutants) chiasmata are not formed, which can be detected by the presence of 12 DAPI-stained bodies, *i.e.* univalents [6]. When meiotic DSBs are induced but not repaired, chromosomal fragmentation occurs, typically resulting in ≥12 irregularly shaped DAPI-stained bodies at diakinesis [19], [20]. Surprisingly, *com-1* mutant oocytes exhibited a different chromosomal pattern: the diakinesis nuclei contained 1 to 12 DAPI-stained entities [14]. We validated this finding by careful inspection of COM-1-deficient diakinesis nuclei (Figure 1C and 1D). These diakinesis nuclei occasionally showed chromosomal fragments, albeit only in 2% of the oocytes (Figure 3C). We argued that the low frequency of chromosomal fragmentation in *com-1* mutants is inconsistent with a conserved role for COM-1 in SPO-11 removal, given that SPO-11-bound DSBs are refractory to repair. Based on the diakinesis studies we envisaged a different scenario in which *com-1* mutants are able to repair meiotic DSBs, yet do so in an error-prone manner, ultimately resulting in chromosomal aggregates and failed chiasmata formation. Several observations supported this hypothesis: Firstly, unlike *spo-11* mutants, *com-1* mutant oocytes hardly ever contained exactly 12 univalents, which indicated that DSBs were induced. Secondly, all diakinesis nuclei had fewer than 12 DAPI-stained bodies and rarely contained small chromosomal fragments, arguing that most programmed DSBs are repaired. Thirdly, the diakinesis nuclei often contained more than 6 DAPI-stained bodies and frequently exhibited DAPI bodies that morphologically resembled univalents, which implied that chiasmata formation was impaired. Finally, many diakinesis nuclei had fewer than six DAPI-stained bodies, potentially reflecting chromosomal entanglements and/or fusions between non-homologous chromosomes.

### Loss of Ku restores chiasmata formation and viability in com-1 mutant animals

To test if the chromosomal aggregation events in *com-1* mutant oocytes were due to inappropriate NHEJ activity, we crossed *com-1* mutants with worms lacking the NHEJ factor CKU-80. Strikingly, *cku-80* deficiency led to a >20 fold increase in viability among *com-1* mutant progeny: while *com-1* single mutants produced 0–2% viable embryos, *com-1 cku-80* double mutants produced 30–40% viable progeny (Figure 1B). Moreover, nearly all hatchlings of *com-1 cku-80* double mutants successfully developed into adults, while *com-1* single mutant hatchlings typically died as arrested L1/L2 larvae.

To verify these observations we crossed animals carrying another allele of *com-1* to worms lacking the other well-conserved Ku subunit CKU-70. The resultant *com-1(t1489) cku-70* double mutants showed identical phenotypes as the aforementioned *com-1(t1626) cku-80* double mutants, including elevated embryonic survival and restored larval development as compared to *com-1(t1489)* single mutants (Figure S1). We therefore conclude that *com-1* deficient animals suffer from toxic Ku activity and that in the absence of Ku, COM-1 is dispensable for *C. elegans* development and gametogenesis.

In contrast to the diakinesis nuclei of *com-1* single mutants, which hardly ever contain six DAPI-stained bodies, 70% of diakinesis nuclei of *com-1 cku-80* double mutants had the wild-type set of six bivalents (Figure 1C and 1D). We obtained similar results for *com-1 cku-70* double mutants (Figure S1). The fact that Ku deficiency restored bivalent formation in *com-1* mutant animals implies that both the univalents and the chromosomal aggregates in *com-1* deficient oocytes were due to Ku-mediated NHEJ. These observations also demonstrate that COM-1 is not required for chiasma formation *per se*. Notably, both bivalent formation and embryonic viability in *com-1 cku-80* double mutants were completely *spo-11*-dependent (Figure 1), which indicates that chiasma formation in *com-1* mutants occurs at programmed DSBs and not at spontaneous DSBs.

Based on these diakinesis studies we conclude that i) COM-1 is crucial to prevent NHEJ activity in meiotic cells; ii) Ku can act efficiently on meiotic DSBs (at least when COM-1 activity is perturbed); iii) a *com-1*-independent mechanism exists that is able to convert SPO-11-induced DSBs into proper chiasmata, and iv) in contrast to Sae2/Ctp1 in yeast, COM-1 is not required for SPO-11 removal in *C. elegans*.

### Ku prevents CO formation in com-1 mutant germlines

Since *com-1* single mutants fail to adequately form chiasmata and this defect can be restored by Ku loss (Figure 1), we reasoned that Ku might obstruct CO formation. In *C. elegans*, exactly one CO occurs per homolog pair and these presumptive CO sites can be visualized by specific recruitment of the fusion protein ZHP-3::GFP at late pachytene/diplotene stage [21], [22]. As shown in Figure 2B, wild-type animals had six ZHP-3::GFP foci in nearly all diplotene nuclei. In contrast, *com-1* single mutants on average had only two ZHP-3::GFP foci per diplotene nucleus (Figure 2A and 2B) and often exhibited persistent ZHP-3::GFP localization along the full length of the synaptonemal complex (SC) – a localization pattern characteristic of CO failure [21].

Importantly, loss of *cku-80* alleviated the ZHP-3::GFP localization defect of *com-1* mutant germlines: virtually all diplotene nuclei of *com-1 cku-80* double mutants had the normal complement of six ZHP-3::GFP foci (Figure 2A and 2B). We conclude that COM-1 is not needed for CO formation *per se*, yet COM-1 is essential to prevent interference by Ku and hence is critical for CO assurance.

### The CO defect of com-1 mutants is due to a scarcity of accessible DSBs

We hypothesized that Ku binds DSB ends and blocks DNA end resection and subsequent meiotic recombination. In order to test if the CO defect observed in *com-1* mutants is due to an insufficient number of DSBs available for HR, we subjected these animals to ionizing radiation (IR) to introduce additional DSBs. 70 Gy of IR did not alter the number of COs in wild-type animals: six ZHP-3::GFP foci were present per diplotene nucleus, irrespective of IR treatment (Figure 2C). Strikingly, 70 Gy of IR substantially increased CO formation in *com-1* mutant animals: while mock-treated *com-1* mutants had on average only two ZHP-3::GFP foci per diplotene nucleus, irradiated *com-1* mutants commonly contained six foci (Figure 2C).

Previous studies have shown that IR can increase CO frequencies only when meiotic DSBs are limiting, *e.g.* in *spo-11* mutants [6], [23], [24]. This effect is attributed to CO homeostasis mechanisms that ensure that meiotic cells receive at least one and only one CO per homolog pair [22], [24]. Our results imply that in the absence of *com-1* CO homeostasis mechanisms are still active and encourage the formation of the obligate COs, yet the substrates to do so are limited. A recent dose-response study estimated that 10 Gy of IR resulted in ∼4 DSBs per chromosome pair, which was sufficient to consistently induce six CO foci in *spo-11* animals [23]. We exposed *com-1* mutants to 10 Gy, 50 Gy and 70 Gy of IR and found that only 70 Gy resulted in a robust induction of six ZHP-3::GFP foci (Figure 2 and Figure S2). The observation that 10 Gy of IR was not sufficient to induce six CO foci in *com-1* mutants, suggests that Ku can also hijack SPO-11-independent DSBs. In support of this notion, IR resulted in increased levels of chromosomal aggregation in *com-1* deficient oocytes (Figure 2D) and [14]. Given the relatively high IR dose needed to allow six CO foci to be formed in COM-1-deficient animals, we propose that IR alleviates the CO defect, not because it introduces SPO-11-independent DSBs, but because it can introduce a total number of DSBs that exceeds the capacity of available Ku, leaving a subset of DSBs unblocked and available for HR.

We conclude that both IR treatment and Ku deletion alleviated the CO deficit in *com-1* mutant animals, yet only Ku deletion restored the bias towards HR-mediated DSB repair.

### Loss of LIG-4 does not restore viability of com-1 mutants

In *com-1* mutant animals Ku causes two problems: defective CO formation and chromosomal aggregation. We next set out to determine how Ku exerts these toxic effects. In classical NHEJ, Ku blocks DNA end resection, stabilizes the break ends and recruits the downstream factor LIG-4, which subsequently seals the DSB [2]. To assess if the Ku complex could be toxic independent of LIG-4-mediated ligation, we made *com-1 lig-4* double mutants and compared those to *com-1 cku-80* and *com-1 cku-70* double mutants. Interestingly, unlike *cku-70* and *cku-80*, the introduction of a *lig-4* null allele did not rescue progeny survival of *com-1* mutants (Figure 3B). Since either *lig-4* or *cku-70/cku-80* loss prevents NHEJ, blocking NHEJ *per se* is not sufficient to restore viability in *com-1* mutants. We therefore infer that Ku has toxic activities that are independent of NHEJ-mediated fusion.

Consistent with that notion, diakinesis nuclei of *com-1 lig-4* double mutants often showed more than six DAPI-stained bodies, indicating that CO formation remained perturbed (Figure 3A). While *lig-4* deletion did not restore the CO defect, it did prevent chromosomal aggregation: in contrast to *com-1* single mutants, the diakinesis nuclei of *com-1 cku-80* and *com-1 lig-4* double mutants rarely had fewer than six DAPI-stained bodies (Figure 1C and Figure 3A). These observations indicate that chromosomal aggregation in *com-1* mutants mainly depends on classical NHEJ.

Notably, diakinesis nuclei of *com-1 lig-4* double mutants frequently contained small DAPI-stained fragments, which are indicative of persistent DSBs (Figure 3A and 3C). We next established that these chromosomal fragments were the consequence of defective repair of programmed SPO-11-induced DSBs (and not of spontaneous DSBs): *com-1 lig-4 spo-11* triple mutant animals exhibited 12 intact univalents at diakinesis and no fragmentation (Figure 3A and 3C). Together, these results strongly suggest that in COM-1-deficient animals, Ku promotes LIG-4-mediated fusions and that in the absence of LIG-4 the Ku-bound DSBs remain unrepaired. We therefore propose that COM-1 needs to prevent Ku activity not only because Ku promotes classical NHEJ at meiotic DSBs, but mainly because Ku forestalls meiotic recombination directly.

### Ku acts at early/mid pachytene stage and blocks the formation of RAD-51 foci

**Figure 4:**
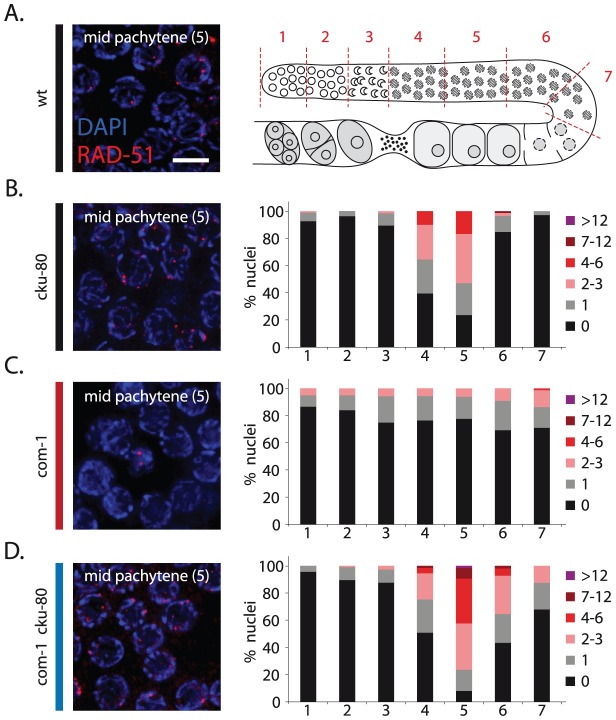
Loss of *cku-80* restores RAD-51 recruitment to meiotic DSBs in *com-1* mutant germlines. (A) Left: representative image of mid-pachytene nuclei in wild-type germlines stained with RAD-51 antibody; merge of RAD-51 (red) and DAPI signal (blue); Right: schematic overview of the C. elegans germline with indicated zones (1–7) used for RAD-51 foci analysis. (B)(C)(D) RAD-51 foci analysis of *cku-80, com-1* and *com-1 cku-80* double mutant germlines, respectively. Left: representative images of mid-pachytene nuclei (zone 5) stained with RAD-51 antibody, merge of RAD-51 (red) and DAPI signal (blue) Right: Stacked histograms depict the quantification of RAD-51 foci in germlines of the indicated genotypes. The number of RAD-51 foci per nucleus is categorized by the color code shown on the right. The percent of nuclei observed for each category (y-axis) are depicted for each zone along the germline axis (x-axis). Three independent gonads were scored for each genotype. Scale bars, 5 µm.

We next determined how and when Ku prevents meiotic recombination. Based on their homologous counterparts, we expect CKU-70/CKU-80 to block DNA end resection. This scenario is consistent with the reported defect in RAD-51 recruitment in COM-1-deficient germlines [14]. Meiotic recombination is initiated in the transition zone where RAD-51-coated recombination intermediates become visible as distinct foci [25], [26]. In wild-type worms, the number of RAD-51 foci peaks at early/mid pachytene stage (Figure 4, zone 4+5) and as repair progresses, these RAD-51 foci disappear by late pachytene stage (Figure 4, zone 6+7) [27]. In *com-1* single mutants, however, we could not detect the typical rise of RAD-51 foci in early/mid pachytene nuclei, suggestive of a defect early in meiotic recombination (Figure 4C, zone 4+5). Strikingly, this defect was relieved by *cku-80* loss: *com-1 cku-80* double mutants did show the strong increase in RAD-51 foci at early/mid pachytene stage (Figure 4D, zone 4+5). These results demonstrate that, in the absence of COM-1, CKU-80 prevents efficient formation of RAD-51-coated HR intermediates, likely by inhibiting DNA end resection. Moreover, they reveal that CKU-80 can already act at early pachytene stage, which paradoxically is the stage where programmed DSBs need to be channeled into HR.

While *com-1 cku-80* double mutant germlines were proficient in RAD-51 loading, we noted a mild delay in RAD-51 focus formation compared to *cku-80* single mutant controls (Figure 4, zone 4+6). This delay suggests that COM-1 may also be required for efficient DNA end resection and thus the timely formation of interhomolog COs.

### COM-1 and EXO-1 act redundantly to promote meiotic recombination

To find the factors responsible for COM-1-independent meiotic recombination, we searched for genes known to have overlapping functions with COM-1 or its homologs. In yeast, the sensitivity of Sae2-deficient mitotic cells to DSB-inducing agents can be rescued by overexpressing the 5′-3′ exonuclease Exo1 [28]. Furthermore, Exo1 transcription is highly induced during yeast meiosis and Exo1 promotes CO formation [29], [30], making Exo1 a suitable candidate for enabling *com-1*-independent CO formation.

**Figure 5:**
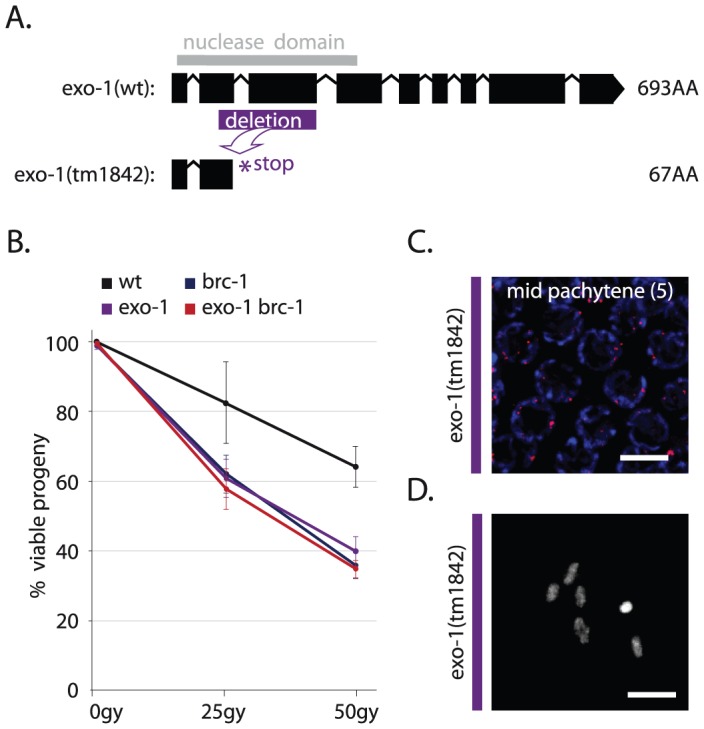
EXO-1 promotes DSB repair in germ cells. (A) Gene model of wild-type F45G2.3 (*exo-1*) with the position of its catalytic domain (gray) and below its truncation allele *tm1842*; a 559 bp deletion (purple) results in a premature stop (B) Percentage progeny survival of animals of the indicated genotype treated with the indicated dose of IR; values are the average of 3 independent experiments, error bars represent S.E.M. (C) RAD-51 immunostaining of mid-pachytene nuclei (zone 5) in *exo-1* deficient germlines; merge of RAD-51 (red) and DAPI signal (blue) (D) Representative picture of a diakinesis nucleus of *exo-1* deficient animals.

A clear Exo1 homolog is present in *C. elegans*, F45G2.3, which we named *exo-1*. We used a deletion mutant of *exo-1*, which is predicted to express a severely truncated protein lacking the conserved nuclease domain (Figure 5A), to show that EXO-1 has a conserved role in HR-mediated DSB repair in germ cells. Firstly, *exo-1* mutant germlines were hypersensitive to IR, in a manner epistatic with the well-studied HR factor *brc-1* (Figure 5B) and secondly, *exo-1* mutants were hypersensitive to transposon-induced DSBs, *i.e. exo-1* deficiency significantly reduced embryonic survival in animals that have elevated levels of transposition in the germline (Figure S3). Despite the need for *exo-1* in repair of ectopic DSBs, unchallenged *exo-1* single mutants did not display major meiotic defects (Figure 5C and 5D), which suggests that EXO-1 does not act on SPO-11-induced DSBs or it operates in a redundant fashion.

**Figure 6:**
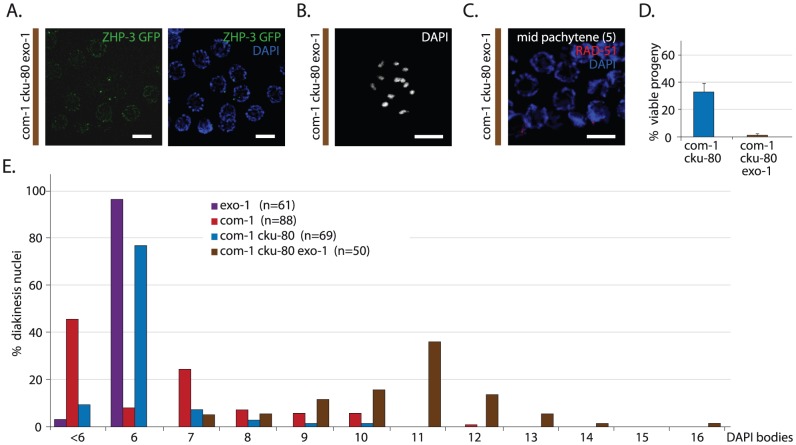
EXO-1 is required for meiotic recombination in absence of COM-1. (A) Representative image of diplotene nuclei of *com-1 cku-80 exo-1* triple mutant animals that express a ZHP-3::GFP transgene (left: GFP signal only, right: merge of GFP and DAPI signal) (B) Representative picture of a diakinesis nucleus in *com-1 cku-80 exo-1* triple mutants germlines (C) RAD-51 immunostaining of mid-pachytene nuclei (zone 5) in *com-1 cku-80 exo-1* mutant germlines; merge of RAD-51 (red) and DAPI signal (blue) (D) Percentage progeny survival of animals of the indicated genotype; values are the average of 3 independent experiments*, error bars represent S.E.M. (E) Frequency distribution of DAPI-stained entities at diakinesis*. n = number of germlines analyzed. The *com-1 cku-80 exo-1* triple mutants occasionally showed >12 DAPI bodies due to chromosomal fragmentation. See Figure 7E for quantification. Scale bars, 5 µm. *These experiments were performed in parallel to those depicted in Figure 1B and 1D; reference values are depicted again here.

To assess if EXO-1 is responsible for COM-1-independent meiotic recombination, we created *com-1 cku-80 exo-1* triple mutants and analyzed CO formation and progeny survival. In contrast to *com-1 cku-80* double mutants, which have robust CO formation (Figure 1C), *com-1 cku-80 exo-1* triple mutants fail to adequately produce COs, as illustrated by the scarcity of ZHP-3::GFP foci at diplotene (Figure 6A) and the lack of chiasmata at diakinesis (Figure 6B). Consequently, *com-1 cku-80 exo-1* animals typically produce aneuploid gametes and hardly any viable progeny (Figure 6D and 6E).

We next investigated how EXO-1 promotes CO formation in *com-1* deficient germlines. Recently, yeast Exo1 has been shown to promote CO formation via two distinct activities: i) by performing DNA end resection and ii) by resolving CO intermediates named double Holliday Junctions (dHJs) [30]. These two Exo1 activities affect HR at different steps: DNA end resection promotes the formation of RAD-51 intermediates, whereas dHJ resolution supports the clearance of RAD-51 intermediates. We found that early/mid pachytene nuclei of *com-1 cku-80 exo-1* triples contained hardly any foci (Figure 6C), which contrasts the many RAD-51 foci observed in *com-1 cku-80* double mutants (Figure 4D). This implies that EXO-1 promotes *com-1*-independent CO formation mainly via its role in DNA end resection.

From these results it can be deduced that i) EXO-1 can act on meiotic DSBs and ii) EXO-1 and COM-1 act in parallel pathways to promote RAD-51 recruitment at early/mid pachytene stage and individually can assure timely CO formation. Furthermore, both COM-1 and EXO-1 are not essential for SPO-11 removal because we did not observe substantial chromosome fragmentation in the diakinesis nuclei of *com-1 cku-80 exo-1* triple mutants. Instead, we detected six to twelve regularly shaped DAPI-stained bodies (Figure 6B and 6E), which suggests some degree of DSB repair.

### Homolog-independent HR does not depend on COM-1 and EXO-1

*C. elegans* germ cells switch between different DSB repair modes as they progress through meiosis [31]. In the early stages of meiotic prophase, the majority of meiotic DSBs are repaired using the homologous chromosome as a template [31], [32]. At late pachytene stage this dominance is thought to be relieved, allowing homolog-independent mechanisms to repair the meiotic DSBs [32], [33]. One example that supports this notion is that mutant animals defective in interhomolog HR (e.g. *syp-2* mutants) show persistent meiotic DSBs that are eventually repaired late in meiotic prophase in a *rad-51*-dependent manner [32]. Subsequent studies suggest that these remaining DSBs are repaired efficiently via intersister HR, ultimately giving rise to intact chromosomes at diakinesis [19], [34].

**Figure 7:**
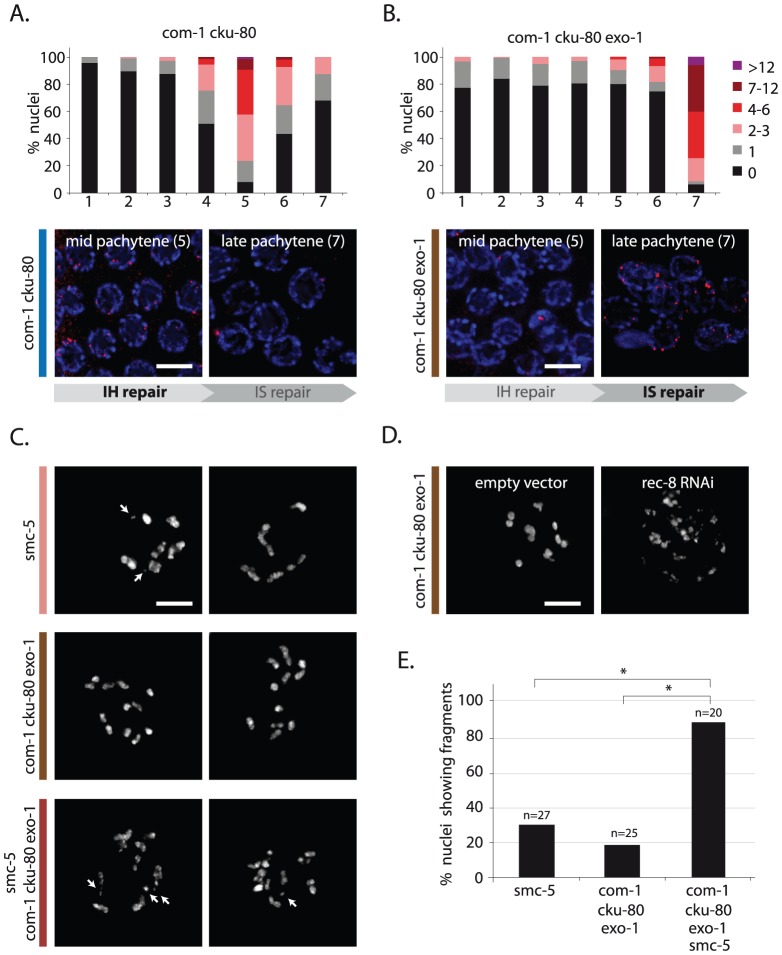
EXO-1 and COM-1 are needed for efficient interhomolog HR, but dispensable for intersister HR. (A,B) RAD-51 foci analysis of *com-1 cku-80* double and *com-1 cku-80 exo-1* triple mutant germlines, respectively. Stacked histograms depict the quantification of RAD-51 foci in germlines of the indicated genotypes. See Figure 4 for details. Representative images of mid-pachytene nuclei (zone 5) and late pachytene nuclei (zone 7) stained with RAD-51 antibody (red). IH = interhomolog, IS = intersister (C) Two representative pictures of diakinesis nuclei of animals of the indicated genotype. White arrows point out chromosomal fragments (D) Representative picture of a diakinesis nucleus *of com-1 cku-80 exo-1* triple mutant animals, which are fed on E. coli strains carrying either a control- or rec-8 RNAi vector. (E) Percentage of diakinesis nuclei that show chromosomal fragments; n = number of germlines analyzed. *The difference between these genotypes was highly significant (p<0.001 by Fisher's exact test, two tailed). Scale bars, 5 µm.

To investigate the contribution of COM-1 and EXO-1 to homolog-independent HR, we quantified RAD-51 focus formation throughout the germline. *com-1 cku-80* double mutants had many RAD-51 foci at early/mid pachytene stage (Figure 7A, zone 4+5), but very few RAD-51 foci at late pachytene stage (Figure 7A, zone 7), indicating that the majority of RAD-51 intermediates were resolved by that point. Conversely, *com-1 cku-80 exo-1* triple mutant germlines had very few RAD-51 foci at early/mid pachytene stage (Figure 7B, zone 4+5), but showed many RAD-51 foci at late pachytene stage (Figure 7B, zone 7). This abundance of RAD-51-coated recombination intermediates at late pachytene implies that COM-1 and EXO-1 are dispensable for DNA end resection at these later stages, which suggests further redundancy and/or temporal regulation of DNA end resection during meiotic prophase. Moreover, these findings imply that intersister HR may not be affected by *com-1* and *exo-1* loss.

To test if intersister HR is responsible for the residual repair activity in the triple mutant, we depleted the cohesin factor REC-8, which is proposed to promote both interhomolog as well as intersister HR [9], [20], [35]. REC-8 depletion caused extensive chromosomal fragmentation in *com-1 cku-80 exo-1* triple mutants (Figure 7D), implying that REC-8-dependent HR is active in the absence of COM-1 and EXO-1. REC-8 depletion, however, has documented pleiotropic effects, including altered SPO-11 activity, which may affect the levels of chromosome fragmentation [31]. We therefore substantiated these findings by deleting Structural Maintenance of Chromosomes 5 *(smc-5)* in *com-1 cku-80 exo-1* animals. *Smc-5* has recently been shown to be specifically required for homolog-independent (presumably intersister) HR during *C. elegans* meiosis [34]. Analogous to REC-8 depletion, deletion of *smc-5* in *com-1 cku-80 exo-1* triple mutants resulted in high levels of chromosome fragmentation at diakinesis (Figure 7C and 7E). Similar results were obtained when deleting the SMC-5 complex partner SMC-6 (Figure S3). Together these observations strongly suggest that, while COM-1 and EXO-1 redundantly promote RAD-51 recruitment and subsequent CO formation at early/mid pachytene stage, at late pachytene stage both proteins are dispensable for RAD-51-mediated intersister HR.

### Ku deficiency does not fully restore genome stability in com-1 mutants

Despite the observation that HR is active and COs are formed in germlines lacking both *com-1* and *cku-80*, progeny survival of *com-1 cku-80* double mutants was not restored to wild-type levels. In fact, ∼70% of *com-1 cku-80* double mutant progeny died during embryonic development (Figure 1B). Moreover, the mutant animals that survived frequently displayed developmental abnormalities, including altered body morphology and faulty vulval development (Figure S4). These phenotypes suggest that Ku-deficient *com-1* mutants still suffered from genomic instability. In support of this notion, *com-1 cku-80* and *com-1 cku-70* double mutants exhibited high levels of X-chromosome non-disjunction, as revealed by a 50-fold increase in XO males among the surviving progeny (Figure S4). Careful analysis of *com-1 cku-80* deficient germlines revealed that the fidelity of meiotic DSB repair is incomplete: diakinesis nuclei of *com-1 cku-80* double mutants occasionally showed chromosomal abnormalities, including unstable bivalent attachments and chromosomal aggregates (Figure 1D and Figure S5). We detected similar chromosomal aberrations in *com-1 lig-4* double mutants (Figure S5), supporting the notion that an alternative mutagenic repair pathway exists that can provoke chromosomal aggregates in germ cells devoid of classical NHEJ [8]. We propose that Ku-deficient *com-1* mutants still suffer from (NHEJ-independent) error-prone repair events, which cause substantial chromosomal instability and embryonic lethality.

We next addressed whether these aberrant repair events in *com-1 cku-80* double mutants induced germ cell apoptosis. Interestingly, despite the high degree of chromosomal instability, the level of apoptosis was not observed to be increased in *com-1* single mutant germlines [14]. Although we cannot formally exclude that COM-1 by itself is required for the signaling of apoptosis, our cytological data argue that Ku blocks end resection in these animals and thus precludes the formation of ssDNA - a major trigger for the DNA damage checkpoint [36], [37]. To test this hypothesis further, we counted apoptotic cells, marked by transgenic CED-1::GFP, in *com-1 cku-80* deficient germlines. We observed a mild but statistically significant increase as compared to *com-1* single mutants (Figure S4). This result may reflect inefficient repair of a fraction of DSBs in *com-1 cku-80* double mutants, as was also suggested by the subtle delay in RAD-51 focus resolution during meiotic prophase (Figure 4, zone 6). These phenotypes are however very mildly different from wild-type behavior [14], [27], [38]. We thus conclude that the vast majority of meiotic DSBs are repaired effectively in *com-1 cku-80* mutant germ cells, without activating the DNA damage checkpoint. The fidelity of repair, however, is clearly affected by *com-1* and *cku-80* loss.

## Discussion

### The conserved C-terminus of COM-1 counteracts Ku activity and thereby supports efficient meiotic recombination

We identified COM-1 as a crucial factor in preventing toxic Ku activity at meiotic DSBs. Both *com-1* alleles used in this study (*t1626* and *t1489*) are loss-of-function alleles and encode for C-terminally truncated proteins [14]. Although sequence analysis of the *t1489* allele revealed a different mutation than previously annotated, both alleles still contain a premature stop that prohibits expression of the conserved C-terminus (Figure S1).

Previously, Penkner and colleagues claimed that COM-1 was required specifically to repair SPO-11-induced DSBs but not IR-induced DSBs – suggestive of a conserved role for COM-1 in SPO-11 removal [14]. However, the data we present here reveals that COM-1-deficient germlines are able to repair SPO-11-induced DSBs both via NHEJ and HR. Moreover, we show that COM-1 is not required for meiotic recombination *per se*, but instead is needed to prevent Ku activity at early pachytene stage to allow DNA end resection and CO formation to take place.

Despite the high conservation of the C-terminal domain of Sae2/COM-1, the contribution of these proteins to SPO-11 removal has clearly diverged between yeast and metazoans: while in yeast a single point mutation in the C-terminus of Sae2 can block Spo11 removal and subsequent HR reactions [39], removal of the entire C-terminus of COM-1 does not prohibit meiotic recombination in *C. elegans*.

**Figure 8:**
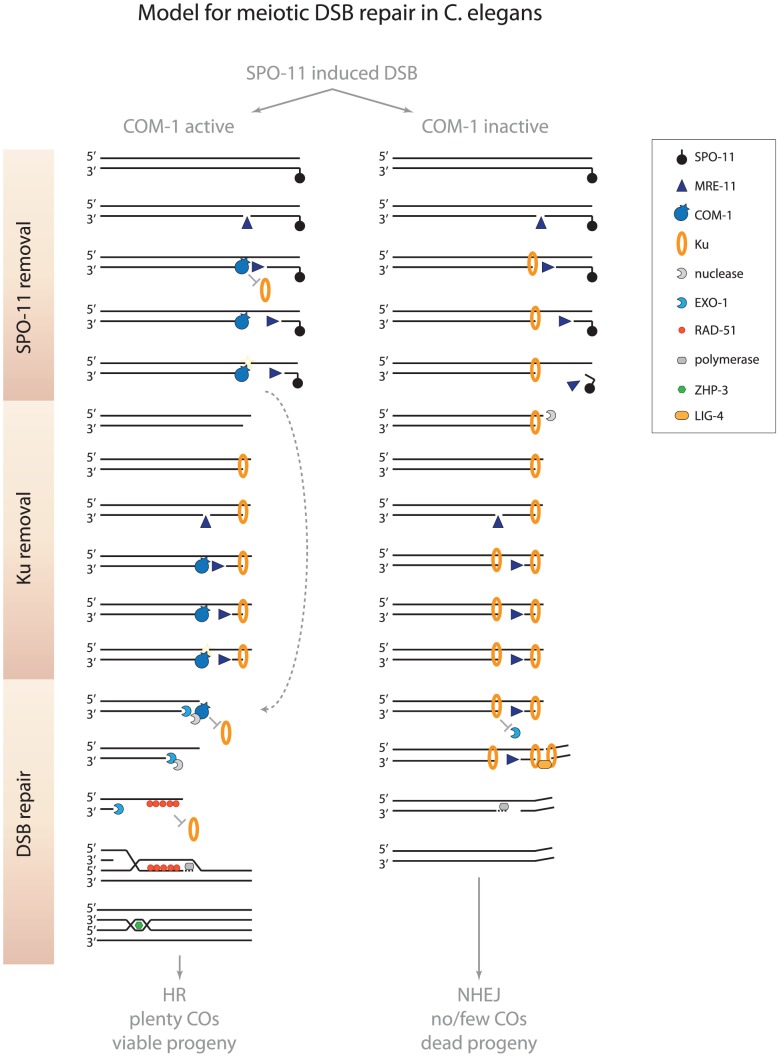
Model for meiotic recombination in *C. elegans*. In wild-type germlines, MRE-11 may create substrates at meiotic DSBs that allow COM-1 to efficiently remove Ku (and SPO-11). When COM-1 function is perturbed, MRE-11 mediated processing may still release SPO-11 bound oligos. However, MRE-11 activity alone is not sufficient to counteract Ku binding and prevent toxic NHEJ activity. Without COM-1 and Ku, SPO-11 is removed and EXO-1 promotes DNA end resection and allows the obligate COs to be formed. See text for further details.

Spo11 removal in yeast not only requires Sae2, but also the highly conserved nuclease Mre11 [18], [40]. Perhaps metazoan MRE-11 is able to remove SPO-11 independently of COM-1. In that scenario, MRE-11 would create free DSB ends that could act as a substrate for both HR and NHEJ (Figure 8). In *C. elegans*, MRE-11 is needed for meiotic DSB formation, however, this requirement can be bypassed by the depletion of meiotic cohesin [41], [42]. Meiotic DSB induction in absence of MRE-11 results in severe chromosome fragmentation, suggesting that MRE-11 is also required for SPO-11 removal in *C. elegans* [42].

We show here that COM-1 is not required for the initiation of meiotic DSB repair, but is needed to channel the programmed DSBs into HR. When COM-1 function is perturbed, Ku blocks EXO-1-mediated resection and promotes LIG-4-mediated fusion. How COM-1 prevents Ku activity on a molecular level is unknown to date, but based on the current models of DNA end resection at meiotic DSBs and the observations described here, we propose that COM-1 cleaves off Ku-bound DSB ends and thereby enables EXO-1 to perform DNA end resection (Figure 8).

### A model for COM-1–dependent Ku removal

Recent work on yeast meiosis has led to a new model for initiation of meiotic recombination that is based on bidirectional DNA end resection [43]. In this model Mre11 creates a single-strand nick up to 300 nucleotides from the meiotic DSB end. This nick then acts as a substrate for both Exo1 and Mre11: Exo1 starts resection in the 5′-3′ direction (away from the DSB) and Mre11 initiates resection in the 3′-5′ direction (towards the DSB end). Accordingly, the 3′-5′ exonuclease activity of Mre11 is critical for the efficient release of Spo11 oligos and subsequent meiotic recombination [43]. Mre11 is proposed to also remove Ku from DSB ends, since Ku (like Spo11) blocks DSB ends and prevents HR-mediated repair [43]. However, recent *in vitro* studies have revealed that human MRE11 cannot compete with Ku for DNA binding nor is able to displace Ku from DSB ends [44]. In these reactions, Ku efficiently prevented EXO1 from performing DNA end resection, even in the presence of MRE11. Our *in vivo* model is consistent with such an interaction, as MRE-11-proficient, but COM-1-deficient, germlines are able to remove SPO-11, but are not able to prevent Ku from hijacking meiotic DSBs (Figure 1 and Figure 2). Our observations imply that SPO-11 removal and Ku exclusion are two distinct activities. Based on the bidirectional nature of DNA end resection and the fact that the affinities of Ku to ssDNA nicks and dsDNA ends are almost equal [45], [46], we propose that Ku may act at the upstream nick to prevent EXO-1-mediated end resection. In such a scenario, MRE-11 may still be able to progress towards the DSB end to remove SPO-11, thus creating a free DSB end that allows NHEJ-mediated repair (Figure 8). The notion that Ku may block 5′-3′ resection by EXO-1, but not 3′-5′ resection by MRE-11 is supported by the fact that the 3′-5′ exonuclease activity of mammalian MRE11 promotes deletion formation during classical NHEJ [47].

We hypothesize that COM-1 prevents Ku occupancy at meiotic DSBs and therefore safeguards proper 5′-3′ DNA end resection and CO formation (Figure 8). While our study reveals that MRE-11, in the absence of COM-1, is not sufficient to prevent Ku activity at meiotic DSBs, we cannot exclude that COM-1 requires MRE-11 activity to counteract Ku. In fact, COM-1 may cut the gapped DNA structure that arises when MRE-11 progresses towards the DSB end, which would release both MRE-11 and Ku from the break site (Figure 8). In support of this model, Sae2 has been shown to possess intrinsic endonuclease activity on gapped DNA substrates *in vitro* and this activity is proportional to the length of exposed ssDNA [48]. Moreover, Sae2 mutants accumulate both Mre11 and Ku at DSB ends [49], [50].

### Ku can act at early pachytene stage and competes with interhomolog HR

Several studies have found evidence of NHEJ-mediated chromosomal aggregates in *C. elegans* germ cells, however, the biological relevance of these NHEJ events has been uncertain, since they were evident only when meiotic recombination was completely abolished (e.g. by *rad-51*, *brc-2* or *msh-4* mutation) and were detected only at diakinesis, the final stage of meiotic prophase [8], [9], [10]. Here we report that Ku can act even when meiotic recombination is proficient and that it does so early in meiosis, at early/mid pachytene stage. Moreover, we reveal that meiotic Ku activity can result in toxic chromosomal aggregates and a fatal lack of obligate COs.

The capacity of Ku to block meiotic recombination is maybe best illustrated by the low levels of ZHP-3::GFP foci observed in COM-1-deficient animals – a phenotype that can be completely alleviated by Ku loss (Figure 2). ZHP-3::GFP localizes to presumptive CO sites and forms six distinct foci in wild-type diplotene nuclei [21]. A recent study by Rosu and colleagues revealed that although each *C. elegans* meiotic nucleus may undergo up to 40 programmed DSBs, a single DSB per chromosome pair is largely sufficient to assure CO formation [51]. Given that more than a third of the COM-1-deficient nuclei are not able to form a single ZHP-3::GFP focus, and the ones that do only form on average 2–3 ZHP-3::GFP foci, we predict that in the absence of COM-1 nearly all meiotic DSBs are blocked by Ku. To shed more light on this subject, we tried to outcompete Ku by creating many extra DSBs using IR. Only when *com-1* mutants were treated with a relatively high dose of IR (estimated to inflict ∼170 DSBs per nucleus [23]) the majority of diplotene nuclei had six CO foci. Based on these experiments we estimate that Ku is able to block ∼97% of all meiotic DSBs when COM-1 function is impaired.

Despite this high toxic capacity of Ku, wild-type worms exhibit very robust CO formation and at least a hundred-fold bias towards HR over NHEJ-mediated repair of germline DSBs [51], [52]. This suggests that COM-1 is very potent in either blocking or removing Ku at meiotic DSBs. Given the striking affinity of Ku towards DNA ends [45] and the detrimental effects of meiotic NHEJ on species survival [this study], additional levels of regulation might be necessary to guarantee the strong HR bias in germ cells. In mouse spermatocytes, Ku protein levels drop significantly at early/mid pachytene stage, revealing that Ku activity can be prevented both by COM-1 activity and at the level of transcription/translation [53]. Interestingly, recently identified COM1 mutants in rice also displayed many non-homologous chromosome entanglements in meiotic cells, indicating that COM-1-mediated NHEJ inhibition may be a common phenomenon among eukaryotes [54].

### COM-1 and EXO-1 promote the timely formation of CO substrates

In addition to its role in NHEJ inhibition, COM-1 also supports DNA end resection during early/mid pachytene stage (Figure 4 and Figure 7). In yeast, DNA end resection at meiotic DSBs is performed by Exo1 and Sgs1/Dna2, with Exo1 having the major role [39]. Accordingly, Exo1 mutants show subtle but significant meiotic defects including reduced spore viability and a two-fold decrease in CO recombination [55]. We show here that COM-1-proficient worms do not rely on EXO-1 for meiotic recombination, as *exo-1* single mutants form both meiotic RAD-51 foci and bivalents normally (Figure 5). When COM-1 is absent however, EXO-1 becomes essential for RAD-51 loading at early/mid pachytene stage and subsequent CO formation (Figure 6). Thus meiotic germ cells require either COM-1 or EXO-1 to perform timely DNA end resection. How COM-1 promotes extensive DNA end resection is still unclear, as COM-1 homologs are implicated only in the onset of resection [49]. COM-1 may be needed for the recruitment of other nucleases to meiotic DSBs. For instance, recruitment of the nuclease DNA2 to DSBs strongly depends on CtIP in human cells [56]. In line with this suggestion, we found a strong synthetic lethal interaction between *exo-1* and *dna-2* (unpublished observations). We also demonstrated that HR via the sister chromatid is not abolished by *com-1* and *exo-1* mutation, revealing another activity that is able to resect meiotic DSBs independent of COM-1 and EXO-1, but only in late pachytene nuclei (Figure 7). Why this activity does not support meiotic recombination and CO formation at early pachytene stage is still an open question.

### Implications of mutagenic NHEJ activity in germ cells

COM-1 is dispensable for meiotic recombination *per se*, however without it, many meiotic DSBs will be repaired via NHEJ, a mutagenic DSB repair pathway that generates non-CO products. The scarcity of COs, combined with the extensive chromosomal aggregation, provides a cogent explanation for the poor fertility of *com-1* mutant animals and reveals the deleterious nature of unscheduled NHEJ during meiosis.

How NHEJ is kept in check during human meiosis remains to be addressed. Recent studies have revealed that a subclass of so-called Seckel and Jawad syndrome patients express truncated CtIP variants that typically lack the conserved C-terminus [57], which are very reminiscent of the *com-1* alleles used for this study (Figure S1). Although these patients suffer from severe mental retardation and skeletal abnormalities, it is unknown to date if they also have fertility defects.

Multicellular animals rely heavily on NHEJ to maintain genome stability in somatic tissues, but the efficacy of this repair pathway seems to come with a price: uncontrolled NHEJ activity has been shown to drive tumorigenesis in mice [58] and the data presented here uncover its toxic properties during meiosis. Recent advances in genome-wide sequencing have revealed that many complex chromosomal rearrangements that occur *de novo* in human germlines show typical NHEJ footprints [59], [60], which suggests that incomplete inhibition of NHEJ during gametogenesis may affect genome evolution in many organisms, including humans, and could lead to pathogenic chromosomal alterations that cause serious inborn diseases.

## Materials and Methods

### Worm strains and culture conditions

All strains were maintained at 15°C using standard *C. elegans* techniques [61]. The wild-type background was Bristol N2. In case of mutant strains that carried a linked *unc-32(e189)* allele, matched *unc-32(e189)* homozygotes served as controls. The following mutations, transgenes and genetic balancers were used:

LGII: *smc-5(ok2421)*, *smc-6(ok329*4), *dna-2* [62], *mln1*[*dpy-10* mIs14].

LGIII: *com-1(t1626)* [14], *com-1(t1489)* [14], *unc-32(e189)*, *cku-80(ok861)*, *cku-70(tm1524)*, *lig-4(ok716)*, *exo-1(tm1842)*, *brc-1(tm1145)*, hT2[*let-? qIs48*].

LG IV: *spo-11(ok79)*, *jfIs2*[ZHP-3::GFP] [21].

### Y-irradiation and progeny survival/him assays

Synchronized L4 worms were either left unchallenged or irradiated using an x-ray generator (200 kV; 10 mA; 11 Gy/min dose rate; YXLON International) to create germline DSBs. Three (irradiated) hermaphrodites were pooled on an OP50 seeded NGM plate and cultured at 20°C to produce progeny. After 40 hrs mothers were removed and the ratio between dead eggs/hatched larvae was assessed 24 hrs later. For Him assay, the percentage of males among the hatched progeny was determined. For all survival/him assays, at least three independent plates were scored per condition. Figures provide mean values of three independent experiments.

### DAPI staining and ZHP-3::GFP analysis

Synchronized L4 worms were picked and allowed to age 20–24 hrs. Gonad dissection was carried out in 1× EBT (25 mM HEPES-Cl pH 7.4, 118 mM NaCl, 48 mM KCl, 2 mM CaCl, 2 mM MgCl, 0.1% Tween 20 and 20 mM sodium azide). An equal volume of 4% formaldehyde in EBT was added (final concentration is 2% formaldehyde) and allowed to incubate for 5 min. The dissected worms were freeze-cracked in liquid nitrogen for 10 min, incubated in methanol at −20°C for 10 min, transferred to PBS/0.1% Tween (PBST), washed 3×10 min in PBS/1%Triton-X and stained 10 min in 0.5 µg/ml DAPI/PBST. Finally samples were de-stained in PBST for 1 h and mounted with Vectashield. Diakinesis nuclei of −1 position oocytes (closest to the spermatheca) were analyzed using Leica DM6000 microscope. To examine CO formation, ZHP-3::GFP foci were analyzed in ∼15 most proximal pachytene/diplotene nuclei of at least six independent germlines (∼100 nuclei).

### Immunofluorescence and RAD-51 focus quantification

RAD-51 protein was detected by indirect immunofluorescence. Germlines were dissected and fixed for whole-mount staining as described above, then blocked with 1% BSA in PBST and incubated overnight at 4°C with rabbit anti-RAD-51 antibody (Novus Biologicals) diluted 1∶200. Primary antibody was detected using Alexa488 Goat anti-rabbit antibody (Invitrogen) diluted 1∶1000 and DNA was counter-stained with 0.5 µg/ml DAPI. RAD-51 foci were imaged using a Leica DM6000 deconvolution microscope collecting 0.5 µm Z-sections. The number of foci per nucleus was counted for each of the seven zones of the germline [27]. Three to five germlines were quantified per condition.

## Supporting Information

Figure S1 Loss of *cku-70* prevents chromosomal aggregation and restores chiasmata formation and embryonic survival in *com-1(t1489)* mutants. (A) Gene model of C44B9.5 (*com-1*) with the position of the non-sense mutations t1626 and t1489 in the third and sixth exon, respectively. Although the annotation of the t1626 allele is correct, the t1489 allele is miss-annotated: no C>T mutation was detected 4030 bp upstream of the ATG (supposedly resulting in an ‘amber’ stop and a 345AA COM-1 peptide). Instead, we found a C>T mutation 4147 bp upstream of the ATG, which leads to a ‘ochre’ stop and a 384AA truncated COM-1 peptide. Notably, both the t1626 and t1489 stops are upstream of the sequence coding for COM-1's well-conserved C- terminal domain. (B) A representative picture of diakinesis nuclei of animals of the indicated genotype. Scale bars, 5 µm. (C) Percentage progeny survival of animals of the indicated genotype; values are the average of 3 independent experiments; error bars represent S.E.M.
(EPS)

Figure S2 Dose-response analysis of ZHP-3::GFP foci formation upon exposure to IR Representative pictures of ZHP-3::GFP foci in diplotene nuclei from either wild-type germlines (left) or *com-1(t1626)* mutant germlines (right), exposed to indicated IR doses. Panels depict, from left to right, DAPI signal with numbers of foci in each nucleus indicated, GFP signal only, and DAPI/GFP merge). Scale bars, 5 µm.
(EPS)

Figure S3 EXO-1 promotes DSB repair in *C. elegans* germ cells. (A) Percentage progeny survival of animals of the indicated genotype. Mut-8 mutation activates transposition in the germline. (B) A representative picture of diakinesis nuclei of animals of the indicated genotype. White arrows point out chromosomal fragments. Scale bar, 5 µm.
(EPS)

Figure S4 Ku deficient *com-1* mutants show various signs of chromosomal instability but only a mild increase in germline apoptosis. (A) Various somatic defects observed in second-generation *com-1 cku-80* double mutants, including dumpy morphology (dpy), egg laying deficiency (egl) and protruding vulvas (pvl). Black scale bar, 50 µm (B) Percentage male progeny of animals of the indicated genotype; values are the average of 3 independent experiments, error bars represent S.E.M. (C) Left: a representative picture of a CED-1:GFP expressing germline (left), with apoptotic cells indicated by white arrows; Right: Average number of apoptotic cells (surrounded by CED-1::GFP) per gonad arm in *com-1(t1626)* and *com-1(t1626) cku-80* mutant animals. Error bars represent SD, n = number of germlines analyzed. *The increase in apoptotic cells was statistically significant (p<0.01 by Student's t-test, two tailed).
(EPS)

Figure S5 NHEJ deficient *com-1* mutants still exhibit chromosomal instability in the germline. (A) Examples of diakinesis nuclei showing chromosomal aberrations in *com-1(t1626) cku-80* mutant germlines. White arrowheads indicate unstable attachments between homologs; white arrows indicate odd-shaped DAPI bodies that may represent chromosomal fusions. Scale bar, 5 µm (B) Examples of diakinesis nuclei showing chromosomal aberrations in *com-1(t1489) lig-4* mutant germlines. White arrows indicate odd-shaped DAPI bodies that may represent chromosomal fusions. Scale bar, 5 µm.
(EPS)
